# Posterior occipito cervical decompression with fixation and fusion in Cranio vertebral junction compression

**DOI:** 10.12669/pjms.335.12988

**Published:** 2017

**Authors:** Lal Rehman, Iram Bokhari, Ali Afzal, Shakeel Ahmad

**Affiliations:** 1 Dr. Lal Rehman, FCPS. Department of Neurosurgery, Jinnah Postgraduate Medical Center, Karachi, Pakistan; 2 Dr. Iram Bokhari, FCPS. Department of Neurosurgery, Jinnah Postgraduate Medical Center, Karachi, Pakistan; 3 Dr. Ali Afzal, MBBS. Department of Neurosurgery, Jinnah Postgraduate Medical Center, Karachi, Pakistan; 4 Dr. Shakeel Ahmed, MBBS. Department of Neurosurgery, Jinnah Postgraduate Medical Center, Karachi, Pakistan

**Keywords:** Craniocervical junction instability, Occipitocervical fusion

## Abstract

**Objective:**

To find out the clinical outcome of posterior decompression with occipitocervical fixation and fusion in patients with Craniovertebral junction instability.

**Methods:**

Eighty consecutive patients of cranio vertebral junction (CVJ) compression were treated in the department of neurosurgery, Jinnah Postgraduate Medical Centre (JPMC), Karachi over a period of 05 years from 1^st^ January 2012 till 31^st^ August 2016. All patients underwent posterior decompression with occipitocervical fusion (OCF) and fixation. The clinical outcome was assessed by Japanese Orthopedic Association (JOA) score and grading.

**Results:**

Out of 80 patients with CVJ instability, 64 (80%) were due to non traumatic causes, while 16 (20%) were secondary to trauma. All 80 patients(100%) showed post operative relief in pain. Sixty four (80%) patients showed improvement in power post operatively while six (7.5%) had no change, four (5%) showed deterioration and six (7.5%) patients expired. Sixty four (80%) patients had improvement of the JOA scores at last follow-up. According to etiology, the JOA score for patients with trauma improved in 12(75%) patients and 52(81.25%) for non traumatic causes while six patients (7.5%) expired. Fusion was achieved in 64 (80%) patients at last follow-up.

**Conclusion:**

Posterior decompression with occipitocervical fusion and fixation is safe and can be recommended in cases of CVJ compression.

## INTRODUCTION

The craniovertebral junction surrounds key neurological structures in a complex arrangement, making instability associated with it, a diagnostic and therapeutic challenge.^1^,^2^ Majority of these cases are associated with non traumatic etiologies like Down’s syndrome, osteogenesis imperfecta, and connective tissue disorders such as Ehlers-Danlos, therefore, special attention should be paid to any member of this cohort exhibiting one or more symptoms.^3^

These injuries may cause immediate fatality or delayed deterioration of neurological function; therefore they require a sound stabilization to be performed as soon as possible.^4^ The exact operative procedure is directed by nature of lesion and degree of compression. The OCF procedure is a technically demanding and severely invalidating surgical procedure, compromising axial rotation of the head above the trunk but also limits flexion–extension. This multi-joint complex allows for >50% of all head and neck movements.^5^ Indication to OCF can only be an instability causing neurologic impairment or a potential neurologic damage. In addition, the sharp angle at which the occiput meets the upper cervical spine creates a significant lever arm that works against surgical fixation devices. Therefore, the most rigid fixation device possible is required to promote fusion.^6^

There has been a lookout for one such surgical procedure which is inherently safe, easily reproducible and biomechanically sound. Thus, this study was done to see the clinical improvements after posterior decompression with occipitocervical fixation and fusion in patients with Craniovertebral junction instability. Hence, this method can be used in patients with this challenging disease entity to reduce its associated morbidity and mortality.

## METHODS

The study was conducted in the department of neurosurgery at Jinnah Postgraduate Medical Centre, Karachi from 1^st^ January 2012 till 31^st^August 2016, after obtaining Institutional Review Board (IRB) approval. Eighty consecutively admitted patients with CVJ compression, 51(63.75%) males and 29 (36.25%) females) were included in the study, secondary to traumatic and non traumatic etiologies as well as neurologically intact patients but with pain or instability on radiology. Patients with malignancy, previous surgery or where only decompression was required were excluded. The specific treatment modality was chosen considering the general medical condition of the patient, severity and location of the fracture, compression of the spinal cord, degree of instability and the neurological status. Pre operative assessment was done via plain x-rays, MRI and 3D CT scans.

All patients were initially given traction and observed clinically and radiologically. In all patients OCF was done either with DCP plates and sublaminar wires of C2 and C3 with occipital bone or occipital plates with C2 pedicle and C3 lateral mass screws where financially feasible. The posterior arch of C1 was resected in all cases. Bone graft was placed between occipital bone and C2 lamina.

X-ray cervical spine and 3D CT scans were done post operatively and on follow-up. The clinical outcome was assessed by JOA score and grading [Table-I]. A hard cervical collar was worn for three months post operatively. The total JOA score and grading assesses motor and sensory functions of four extremities and sphincter, amounting to a total of seventeen points. The lower the score, the more severe the deficits. The JOA score was assessed before the operation, at discharge, one month and finally at six months follow up.

**Table-I:** Japanese Orthopaedic Association Score, JOA Score. (Modified by Keller 1993)

*Criterion*	*Points*
***Motor function***	
Paralysis	1
***Upper extremity***	
Fine motor function massively decreased	2
Fine motor function decelerated	3
Discreet weakness in hands or proximal arm	4
Normal function	5
Motor function Unable to walk	1
***Lower extremity***	
Need walking aid on flat floor	2
Need handrail on stairs	3
Able to walk without walking aid, but inadequate	4
Normal function	5
***Sensory***	
Upper extremity/lower extremity/trunk	
Apparent sensory loss	1
Minimal sensory loss	2
Normal function	3
***Bladder function***	
Urinary retention	1
Severe dysfunction	2
Mild dysfunction	3
Normal function	4

Data was collected with help of performa including history, examination, relevant pre and postoperative radiology (X ray, 3D CT and MRI) and post op clinical findings. Statistical analysis was done using SPSS version 22. Categorical variables were expressed in frequency, whereas continuous or quantitative variables such as patient’s age was expressed in mean with ±Standard Deviation. Chi square test was applied post stratification between two groups of traumatic and non traumatic patient population. P value of ≤0.05 was taken as significant.

## RESULTS

The study comprised of 10 children and 70 adults with a mean age of 24 years ± 12yrs amongst which most were males 51 (63.75%). Out of 80 patients with CVJ instability, 64(80%) of patients were non traumatic secondary to entities like basilar invagination in (20/80, 25%), Down’s syndrome (5/80, 6.25%), tuberculosis (10/80 12.5%) or rheumatoid arthritis (5/80, 6.25%) and others. The remaining 16(20%) patients were traumatic. All patients suffered from severe neck pain or neurological deficit pre operatively.

All 80 (100%) patients showed post operative relief in pain. Out of 80 patients, 64(80%) had improvement of the JOA scores at last follow-up. According to etiology, the JOA score for patients with trauma improved in 12(75%) patients as shown in [Table-II] and 52(81.25%) for non traumatic causes [Table-III]. Fusion was achieved in 64 (80%) patients at last follow-up. This study showed statistically significant improvement in neurological status, as assessed by JOA scoring in 64 (80%) patients (p-value ≤0.05) post operatively while four (5%) patients had no change, four (5%) patients showed deterioration [Table-IV]. Six (7.5%) patients expired, four due to respiratory compromise and two due to pneumonia and sepsis.

**Table-II:** Non traumatic.

*Pre operative*		*Post operative*				

(n=64)		Normal Function	Grade 1	Grade 2	Grade 3	Expired
Normal Function	2	1	1			
Grade 1	18	15	1	0	1	1
Grade 2	30	12	15	1	1	1
Grade 3	14	0	2	7	2	3
TOTAL	64	28	19	8	4	5

**Table-III:** Traumatic.

*Pre operative*		*Post operative*					

(n=16)		Normal Function	Grade 1	Grade 2	Grade 3	Expired	Total
Normal Function	1	1					1
Grade 1	3	3	0				3
Grade 2	8	4	2	1	1		8
Grade 3	4	0	0	1	2	1	4
TOTAL	16	8	2	2	3	1	16

**Table-IV:** Surgical Outcome.

*Outcome(80)*	*Traumatic(16)*	*Nontraumatic(64)*	*Total*
Improved (n=64)	12	52	64 (84%) ; p value: 0.03
No change (n=6)	2	4	6 (0.075%)
Deteriorated (n=4)	1	3	4 (0.05%)
Expired (n=6)	1	5	6 (0.075%)

## DISCUSSION

Instability of the CVJ imposes diagnostic and therapeutic problems due to its complex anatomy and biomechanical characteristics. These injuries may cause immediate fatality or delayed deterioration of neurological function.^7^ Lesions of CVJ with cervicomedullary compression are associated with high morbidity and mortality rates. Thus, the aims of occipitocervical fusion surgery are to restore normal alignment, to ensure adequate decompression and to achieve structural stability. Presentation varies from progressive myelopathy, radiculopathy, lower cranial nerve dysfunction, or deformities of the craniocervical region. Operative stabilization of the craniocervical junction is the most common treatment described in the literature^8^,^9^ and in similar manner, we have done fixation and stabilization in all our patients. In patients with such instability, the indication for fusion is an extensive posterior instrumentation fixation procedure that sacrifices the motion of the occipital and C 1, 2 complex and is still controversial.

Several clinical measures of disease severity have been developed such as the Japanese Orthopaedic Association (JOA), Nurick, and Chile’s modified Japanese Orthopaedic Association (mJOA) scoring systems.^10^ These scales enable the clinician to quantify and assess the extent and progression of the disease. We used JOA grading system in our series. The goals of fixation were to achieve anatomic alignment, protect neural elements, stabilize the spine while preserving the normal motion of elements and to produce a “functional decompression”. Significant (70.2% of patients) improvement of the JOA scores was noticed after surgery in our patients and the results are consistent with the 75% to 95% improvement rate reported in literature.^10^-^12^ Based on existing literature, techniques using screw/rod constructs in occipitocervical fusion are associated with very favorable outcomes,^13^ similar to our study where we have rods and screws construct.

Diverse OCF techniques such as screw-rod and occipito-cervical hook are currently available, and all are shown to have high fusion rates (89-100%).^14^ Screw-rod fixation allows for strong biomechanical fixation, immediate stability after surgical fixation with no additional external fixation. Statistically significant neurologic improvement (recovery rate >50%) was demonstrated by 12(75%) of patients in trauma and 52(81.25%) in non traumatic cases, comparable to Galbraith et al.^15^ who report a 30% to 40% significant improvement in myelopathy.

Many researchers have discussed postoperative fusion rates in CVJ patients undergoing occipitocervical or atlantoaxial arthrodesis. Fusion rate of the CVJ lesion is remarkably successful as reported from 75% to 100% and our results at 80% lie within this range.^16^ Nearly all studies have demonstrated consistently high fusion rates with cervicovertebral junction fixation regardless of fusion methods and underlying pathology.^17^ Modern case reports, however, have documented improved neurological outcomes, likely as a result of earlier diagnosis and surgical stabilization.^18^

Extreme difficulties are encountered in paediatric age group because of less developed anatomy, fragile bones and more surgery related complications in children as compared to adult group. However, good outcome was observed in our series of patients as only one out of ten patients deteriorated neurologically in comparison to three adult patients in our study. Probably, advancements in emergency care and diagnostic methods have increased the number of children who survive atlanto-occipital dislocation.^19^ It is further recommended that if there is neurological decline after fixation, obstructive hydrocephalus should be suspected. Four patients (one child and three adults) showed neurological deterioration. These were due to difficulties in intubation, failure to achieve adequate decompression and infection. Mortality in our series was 7.5% in which four were due to respiratory compromise and two due to pneumonia and sepsis. This was comparable with other studies like Song et al.^7^ at 3% and Cappuccio et al. at 16.67%.

Recent biomechanical studies have shown that the screw-based construct is more rigid than the wire-rod construct and reduced the need for external orthoses, but it is rather unclear whether it will result in a higher rate of fusion. Reports of fusion rate of 80% after wiring and of 94% after screwing do not appear to influence markedly in final clinical results.^20^

## CONCLUSION

Posterior decompression and occipitocervical fusion is safe with a high percentage of favorable outcomes and to be recommended in cases of CVJ compression. We recommend that the technique of CVJ fixation should be individualized based on the location and extent of the injury. However, for a successful outcome, careful patient selection and accurate imaging diagnosis are essential.

### Authors` Contribution

**LR** did review and final approval of manuscript.

**IB** did statistical analysis & editing of manuscript.

**AA** did data collection and manuscript writing.

**SA** conceived and designed the study.
